# Severe Euglycemic diabetic ketoacidosis with mixed Acid–Base disorder following acetazolamide initiation in type 2 diabetes: a case report and review of literature

**DOI:** 10.1093/omcr/omag058

**Published:** 2026-05-10

**Authors:** Saif Khaled Abdalhadi Azzam, Lina Barhoum Barhoum, Salih AbuRajab, Nagham Khaled Abdalhadi Azzam, Youssef Abdelqader Alsaeed, Rostom Karajeh, Musad Shawar

**Affiliations:** Department of Clinical Medical Sciences, Faculty of Medicine and Health Sciences, Palestine Polytechnic University, Wadi Al-Harieh Street, Hebron District, Hebron, West Bank P720, State of Palestine; Department of Clinical Medical Sciences, Faculty of Medicine and Health Sciences, Palestine Polytechnic University, Wadi Al-Harieh Street, Hebron District, Hebron, West Bank P720, State of Palestine; Department of Clinical Medical Sciences, Faculty of Medicine and Health Sciences, Palestine Polytechnic University, Wadi Al-Harieh Street, Hebron District, Hebron, West Bank P720, State of Palestine; Department of Clinical Medical Sciences, Faculty of Medicine and Health Sciences, Palestine Polytechnic University, Wadi Al-Harieh Street, Hebron District, Hebron, West Bank P720, State of Palestine; Anesthesiology and Critical Care Department, Palestinian Red Crescent Hospital, Al Salam Street, Hebron, West Bank P720, State of Palestine; Anesthesiology and Critical Care Department, Palestinian Red Crescent Hospital, Al Salam Street, Hebron, West Bank P720, State of Palestine; Anesthesiology and Critical Care Department, Palestinian Red Crescent Hospital, Al Salam Street, Hebron, West Bank P720, State of Palestine

**Keywords:** euglycemic diabetic ketoacidosis, SGLT2 inhibitors, acetazolamide, mixed acid–base disorder, type 2 diabetes

## Abstract

Euglycemic diabetic ketoacidosis (euDKA) is an increasingly recognized complication of sodium-glucose cotransporter-2 (SGLT2) inhibitor therapy, characterized by severe metabolic acidosis with near-normal blood glucose levels. We report a rare case of a 45-year-old woman with type 2 diabetes mellitus who presented with severe euDKA (pH 6.85, glucose 170 mg/dl) following initiation of acetazolamide for glaucoma management. Clinical evaluation revealed profound metabolic acidosis with bicarbonate 1.5 mmol/l and anion gap 27 mmol/l. Delta ratio calculation (0.64) revealed a mixed high anion gap and normal anion gap metabolic acidosis, indicating dual pathophysiology: ketoacid accumulation from euDKA and acetazolamide-induced renal bicarbonate wasting. Immediate acetazolamide discontinuation combined with aggressive fluid resuscitation, insulin infusion, and sodium bicarbonate resulted in complete metabolic correction by Day 3 without hemodialysis. To our knowledge, this rare combination of SGLT2 inhibitor use and acetazolamide underscores the need for careful medication reconciliation and multidisciplinary communication in diabetic patients to prevent complications.

## Introduction

Euglycemic diabetic ketoacidosis (euDKA) is an atypical presentation of diabetic ketoacidosis characterized by severe metabolic acidosis with blood glucose levels below 200 mg/dl [1]. The incidence of euDKA has increased substantially with widespread use of sodium-glucose cotransporter-2 (SGLT2) inhibitors in type 2 diabetes [1]. SGLT2 inhibitors promote urinary glucose excretion, triggering ketogenesis without proportional hyperglycemia, making diagnosis challenging [1].

Acetazolamide, a carbonic anhydrase inhibitor prescribed for glaucoma, causes type 2 renal tubular acidosis through bicarbonate wasting [2, 3]. To our knowledge, the concurrent use of acetazolamide with SGLT2 inhibitors creating mixed acid–base disorder has not been previously reported. We present a rare case of severe euglycemic DKA with mixed high anion gap and normal anion gap metabolic acidosis in a 45-year-old woman with type 2 diabetes precipitated by acetazolamide for glaucoma, highlighting the importance of multidisciplinary medication reconciliation.

## Case report

A 45-year-old woman with four-year history of type 2 diabetes mellitus complicated by diabetic retinopathy presented to the emergency department with three-day history of progressive decline in consciousness. Following an emotional stressor, she developed fatigue, nausea, markedly decreased oral intake, dysarthria, dizziness, and progressive dyspnea. She had no previous history of diabetic ketoacidosis or similar metabolic decompensation.

Her medications included empagliflozin (25 mg daily), semaglutide, insulin glargine, rosuvastatin, and acetazolamide, recently prescribed by her ophthalmologist for glaucoma management.

On examination, the patient appeared acutely ill and profoundly dehydrated. She was conscious but disoriented to time and place. Vital signs revealed tachycardia and marked tachypnea (respiratory rate 30–36 breaths/minute). Chest was clear on auscultation. Neurological examination demonstrated globally decreased motor power (4/5 in all limbs).

Laboratory investigations revealed random blood glucose of 170 mg/dl, classifying this as euglycemic diabetic ketoacidosis. Complete blood count showed leukocytosis (18.85 × 10^9^/l), hemoglobin 15.53 g/dl, and platelets 328.9 × 10^9^/l. Serum biochemistry demonstrated sodium 139 mmol/l, potassium 4.2 mmol/l, and chloride elevated at 111 mmol/l (reference: 99–110 mmol/l). Serum creatinine was 0.77 mg/dl, indicating preserved renal function, and serum lactate was 14.6 mg/dl, not suggestive of lactic acidosis.

Arterial blood gas analysis revealed profound metabolic acidosis with pH 6.85, bicarbonate 1.5 mmol/l, pCO₂ 9.4 mmHg, and anion gap 27 mmol/l. Base excess was −31 mmol/l. Urinalysis showed glucose 3+ and ketones 3 +.

The delta ratio, calculated as (anion gap—12)/(24—HCO₃^−^) = 0.64, was diagnostic of mixed high anion gap and normal anion gap metabolic acidosis, indicating two concurrent processes: ketoacid accumulation (DKA) and acetazolamide-induced renal bicarbonate wasting. The elevated chloride supported hyperchloremic acidosis. Serial measurements throughout hospitalization are detailed in Table 1.

**Table 1:** Serial laboratory and arterial blood gas parameters from admission to discharge.

Time since admission (hours)	PH	HCO3- (mmol/L)	Na/ Cl (mmol/L)	K+ (mmol/L)	Anion gap (mmol/L)	delta ratio
0 h	6.85	1.5	139/111	4.2	26.5	0.64
4 h	6.87	1.4	140/111	3.9	27.6	0.69
8 h	6.92	2.6	147/118	3.1	26.4	0.67
12 h	7.11	6.2	149/122	3.4	20.8	0.49
16 h	7.15	4.1	151/127	3.5	19.9	0.4
20 h	7.16	3.3	144/120	3.1	20.7	0.42
24 h	7.15	7.4	143/117	3.7	18.6	0.4
36 h	7.3	10	136/113	2.8	13	0.04
48 h	7.38	20.4	140/112	3.4	7.6	-1.22
60 h	7.4	22	140/112	3.6	6	-3
72 h	7.41	22.2	140/109	4	8.8	-1.78

Brain imaging revealed old ischemic changes without acute pathology. Transthoracic echocardiography was normal.

The patient was admitted to intensive care. Acetazolamide was immediately discontinued. Management included intravenous fluids with normal saline and 5% dextrose, continuous insulin infusion (2–3 units/h), sodium bicarbonate (75 mEq in 524 ml 5%dextrose over 8 h, repeated for pH < 6.9), potassium chloride, and empirical antibiotics, although cultures remained negative and no infection was confirmed.

Serial arterial blood gas measurements were monitored (Table 1). Over the first 24 h, pH improved from 6.85 to 7.00–7.15, with bicarbonate rising to 4.1–7.4 mmol/l. The patient received 7580 ml intravenous fluids with 5050 ml urine output. By Day 2, pH reached 7.27–7.34. By Day 3, complete normalization was achieved with pH 7.39–7.41 and bicarbonate 22.0–22.2 mmol/l. The patient became fully alert and was transferred from ICU. Insulin was transitioned to subcutaneous insulin glargine 18 units daily.

On Day 4, the patient was discharged home in excellent condition. Discharge medications included levofloxacin, metronidazole, insulin glargine 18 units daily, aspirin 100 mg daily, and rosuvastatin 20 mg daily. Empagliflozin was permanently discontinued. The patient was instructed to monitor blood glucose daily and follow up within one week.

At one-week follow-up, the patient remained completely asymptomatic with no recurrence and excellent glycemic control.

## Discussion

Euglycemic diabetic ketoacidosis (DKA) is an increasingly recognized complication of sodium-glucose cotransporter-2 (SGLT2) inhibitor therapy, characterized by significant ketonemia and metabolic acidosis despite blood glucose levels below 200 mg/dl [1, 4]. Our patient’s profound acidosis (pH 6.85) with a glucose level of 170 mg/dl illustrates how reliance on marked hyperglycemia can delay recognition of this entity in clinical practice [1]. SGLT2 inhibitors promote urinary glucose excretion while increasing the glucagon-to-insulin ratio, thereby stimulating ketogenesis without proportional hyperglycemia [1].

The defining hallmark of this case was recognition of a mixed high anion gap and normal anion gap metabolic acidosis, confirmed through delta ratio calculation [5]. The delta ratio of 0.64 (less than 1.0) indicated that bicarbonate decline exceeded anion gap rise, proving an additional bicarbonate-losing process beyond ketoacid accumulation [5]. This finding directed attention to acetazolamide, which inhibits carbonic anhydrase in the proximal tubule, preventing bicarbonate reabsorption and causing type 2 renal tubular acidosis [2, 3]. The elevated serum chloride (111 mmol/l) provided confirmatory evidence of hyperchloremic acidosis [2, 3].

The combination of empagliflozin and acetazolamide created a dangerous synergistic effect on acid–base balance, with both medications promoting bicarbonate loss through distinct mechanisms [2–4, 6]. To our knowledge, this specific combination has not been previously reported in the medical literature. Pandit et al. reported a similar case involving type 1 diabetes and high-dose acetazolamide (2000 mg) for altitude sickness requiring hemodialysis [6]. However, our case differs significantly: type 2 diabetes, therapeutic-dose acetazolamide for glaucoma, concurrent SGLT2 inhibitor use, and successful medical management without dialysis [1, 4].

Management required a multifaceted approach addressing both acidosis components [1, 7]. Immediate acetazolamide discontinuation was essential to halt bicarbonate wasting [2, 3]. Sodium bicarbonate administration was justified given the extreme pH (<6.9), which carries risk for life-threatening cardiovascular complications [7]. Early dextrose-containing fluids prevented hypoglycemia during insulin therapy, a critical distinction from classic DKA management [1].

This case underscores the importance of comprehensive medication reconciliation and multidisciplinary communication in diabetes care [1]. Clinicians must maintain heightened awareness of euglycemic DKA as an emerging SGLT2 inhibitor complication and exercise caution when prescribing additional medications affecting acid–base balance, particularly acetazolamide, in diabetic patients. Delta ratio calculation proved invaluable for recognizing the mixed acidosis and guiding appropriate therapeutic intervention [5]. Although quantitative β-hydroxybutyrate and measured serum osmolality were unavailable, the normal lactate and creatinine values and the overall clinical and biochemical course support our diagnosis.
